# Supplementary material for: Combined associations of body mass index and adherence to a Mediterranean-like diet with all-cause and cardiovascular mortality: A cohort study
Source: PLoS Med. 2020 Sep 17;17(9):e1003331. doi: 10.1371/journal.pmed.1003331 (PMC7497998; doi:10.1371/journal.pmed.1003331)
Supplement: S1 Protocol — (DOCX) [file pmed.1003331.s002.docx]

**S1 Protocol**. Prospective study plan (in Swedish) [dx.doi.org/10.17504/protocols.io.bgftjtnn](https://dx.doi.org/10.17504/protocols.io.bgftjtnn)

Analysplan BMI, kost och mortalitet

## Övergripande frågeställning

Skiljer sig associationen mellan BMI och mortalitet för olika nivåer av medelhavskost?

## Data

Grunddata är de SMC och COSM 1997 (01 jan 1998), samt 2008/2009 (14 apr 2009). På detta lägger vi till information från patientregistret och dödsregistret.

## Studiestart

1998-01-01

## Exklusion

Vi exkluderar de med BMI<20 vid baseline. De som ännu inte fått utfallet vid 2009, men som har en BMI<20 vid 2009 kommer att censureras 14 apr 2009.

## Primärt utfall

### Tid till död

| **Typ av död** | **Variabelnamn** | **ICD10** | **ICD9** |
| --- | --- | --- | --- |
| Generell död | general_death |  |  |

## BMI

bmi (kg/m^2^) och bmi_cat (20-25, 25-30, 30-)

## Kostmönster

Mediterranean Diet Score (mds) beräknas med samma komponenter som ”Mediterranean Diet and Hip Fracture in Swedish Men and Women” (doi: 10.1002/jbmr.2896). För beräkning av score för samtliga komponenter förutom alkohol och olja använder vi medianerna från 1997-data (även för beräkning av score 2009). Den totala scoren beräknas sedan på två sätt. Dels på ett diskret (den matematiska betydelsen av ordet, motsatsen till kontinuerlig) där varje komponent kan ge 0 eller 1 poäng. Dels på ett mer kontinuerligt sätt, där varje komponent utom olja kan ge 0 till 1 poäng. Olja är fortfarande binär. Beräkningen av dessa kontinuerliga poäng görs i linje med Knudsen et al (2012, DOI: <https://doi.org/10.3402/fnr.v56i0.17129>).

Formeln som användes för de komponenter med en lägre gräns (fv, baljnöt, grains, fish, ferment) var:

Om komponenten är <= median_1997_: Komponent/median_1997_

Om komponenten är > median_1997_: 1

Formeln som användes för kött (med en övre gräns) var:

Kött <= median_1997_: 1

median_1997_ < Kött <= 2 × median_1997_: 1 - (Kött - median_1997_)/median_1997_

Kött > 2 × median_1997_: 0

Formeln som användes för alkohol (5-15 g) var:

Om alkohol 0-5 g                      -> Alkohol/5 poäng

Om alkohol 5-15 g                    -> 1 poäng

Om alkohol 15-30g                  -> 1 - (Alkohol-15)/15 poäng

Om alkohol >30g                      -> 0 poäng

Den trikotomiserade versionen av den diskreta MDS-variabeln heter mds_disc_tri, med kategorier 0-3, 3-5, 6-8. Den trikotomiserade versionen av den kontinuerliga MDS-variabeln heter mds_cont_tri, med kategorier 0-<4, 4-<6, 6-8.

### Tidsinvarianta kovariat

| **Variabel** | **Information** | **Variabelnamn** |
| --- | --- | --- |
| Age at start of study | Födelsedatum finns i SMC1987 | age |
| Educational level (≤9, 10-12, >12 years, other) | Välj det högsta värdet av alla enkäter | educat |
| Charlson comorbidity index (continuous) | Vid baseline | cci_unweighted  cci_weighted |
| Diabetes | ICD-9: 250, ICD-10: E10-E14 Vid baseline | diabetes |

### Tidsvarierande kovariat (gråmarkerade finns i data, men förväntas inte användas speciellt mycket)

| **Variabel** | **Information** | **Variabelnamn** |
| --- | --- | --- |
| Total energy intake (continuous) |  | energy_total |
| Height (continuous) |  | height |
| Physical activity (five categories) |  | exercise |
| Walking/cycling |  | walk |
| Smoking status (never, former, current) |  | smoke |
| Living alone |  | alone |
| Protein intake | Inte justera för denna i huvudanalysen | eprotein |
| Weight (continuous) | Finns i data, men är inte aktuell för användning. | weight |
| Calorie adjusted retinol |  | eretinol |
| Vitamin D intake |  | evitd |
| Alcohol |  | alcohol |
| Cortisone | Inte justera för denna i huvudanalysen | cortisone |
| Saturated fat | Inte justera för denna i huvudanalysen | sfat |

## Analyser

1. Deskriptiv data
2. Poolat data. Använd kohort (kön) som strata. Interaktionsvariabeln mellan bmi_cat och mds_cont_tri genererar nio kategorier. Använd kategori (BMI<25; MDS>=6) som referens. Justera för age, energy_total, height, cci_weighted (endast baseline), diabetes (endast baseline), educat, exercise, walk, smoke, alone.
3. Som 2. fast kontinuerlig version av mds_cont_tri och bmi, utan interaktion.
4. Som 2. fast undersök kring heterogeneitet för MDS-BMI variabeln mellan kön.
5. Som 2. fast könsspecifikt. Generera heatmaps.
6. Stratifierade analyser utan interaktion mellan MDS och BMI. Generera två forest plots. Behandla MDS kontinuerligt linjärt, och BMI kontinuerligt linjärt, med olika HR för BMI<26 respektive BMI>26.
7. Som 2., fast endast åldersjustering.
8. Complete case, där missing vid 2008/2009 innebär last observation carried forward.
9. Som 2., fast begränsa analysen till BMI>22.
10. Ålderstandardiserad descriptives.
11. Complete case (som 8.) fast justera även för ”packyears”
12. Som 2., fast begränsa analysen till BMI≤35.
13. Som 2., fast uppdatera inte variablerna 2009.
14. Som 2., fast starta klockan 2009.
15. Som 2., fast inkludera endast ”never smoker” vid baseline, och censurera därefter ”ever smoker” 2009.

**S1 Protocol**. Prospective study plan (English translation)

Analysis plan BMI, diet and mortality

## Primary objective

Does the association between BMI and mortality differ by different levels of adherence to a Mediterranean diet?

## Data

Baseline data were those from SMC and COSM in 1997 ( baseline used January 1^st^, 1998), with time-updated information from 2008/2009 (April 14^th^, 2009). In addition we use information from the national Patient register and the Cause of Death register.

## Study entry

1998-01-01

## Exclusion

We excluded those with BMI<20 kg/m^2^ at baseline. Those had not had the outcome in 2009 but who had reported a BMI<20 in 2009 will be censored at April 14^th^, 2009.

## Primary outcome

### Time to death

| **Death category** | **Variable name** | **ICD10** | **ICD9** |
| --- | --- | --- | --- |
| All-cause mortality | general_death |  |  |

## BMI

bmi (kg/m^2^) och bmi_cat (20-25, 25-30, 30-)

## Dietary pattern

Mediterranean Diet Score (mds) is estimated with use of the same components as in ”Mediterranean Diet and Hip Fracture in Swedish Men and Women” (doi: 10.1002/jbmr.2896). For the calculation of the score, the median in 1997 is used for all components except for alcohol and vegetable oil (the same medians are used for the score in 2009). The total score is then calculated in two ways, one with where each component is given a value of 0 or 1 and one with where each component has a continuous value between 0 to 1. In the latter calculation, oil is still used as a binary exposure. Calculations of these continuous scores are done according to the method described by Knudsen et al (2012, DOI: <https://doi.org/10.3402/fnr.v56i0.17129>).

The formula used for the components with a lower risk threshold (fruits and vegetables, legumes and nuts, grains, fish, fermented dairy) was:

Component value <= median_1997_: Component/median_1997_

Component value > median_1997_: 1

The formula used for meat (with an upper risk limit) was:

Meat <= median_1997_: 1

median_1997_ < Meat <= 2 × median_1997_: 1 - (Meat - median_1997_)/median_1997_

Meat > 2 × median_1997_: 0

The formula used for alcohol (5-15 g) was:

If Alcohol 0-5 g                      -> Alcohol/5 points

If Alcohol 5-15 g                    -> 1 poäng

If Alcohol 15-30g                  -> 1 - (Alcohol-15)/15 points

If Alcohol >30g                      -> 0 poäng

The trichotomized version of the discrete MDS variable is named mds_disc_tri, with category cutoffs of 0-3, 3-5, 6-8. The trichotomized version of the continuous MDS variable is named mds_cont_tri, with category cutoffs 0-<4, 4-<6, 6-8.

### Covariates not time-updated

| **Variabel** | **Information** | **Variabelnamn** |
| --- | --- | --- |
| Age at start of study | Date of birth is found in SMC1987 | age |
| Educational level (≤9, 10-12, >12 years, other) | Use highest degree from the questionnaires | educat |
| Charlson comorbidity index (continuous) | At baseline | cci_unweighted  cci_weighted |
| Diabetes | ICD-9: 250, ICD-10: E10-E14 at baseline | diabetes |

### Time-updated covariates (variables in grey are to be found in the data set but are not suspected to be used)

| **Variabel** | **Information** | **Variabelnamn** |
| --- | --- | --- |
| Total energy intake (continuous) |  | energy_total |
| Height (continuous) |  | height |
| Physical activity (five categories) |  | exercise |
| Walking/cycling |  | walk |
| Smoking status (never, former, current) |  | smoke |
| Living alone |  | alone |
| Protein intake | Not adjusted for in the main analysis | eprotein |
| Weight (continuous) | Existing in the data set but is relevant for the present analysis. | weight |
| Calorie adjusted retinol |  | eretinol |
| Vitamin D intake |  | evitd |
| Alcohol |  | alcohol |
| Cortisone | Not adjusted for in the main analysis | cortisone |
| Saturated fat | Not adjusted for in the main analysis | sfat |

## Analyses

1. Descriptives
2. Pooled data. Use cohort (sex) as strata. Cross-classification between bmi_cat and mds_cont_tri genererates nine categories. Use category (BMI<25; MDS>=6) as the reference. Adjust for age, energy_total, height, cci_weighted (only baseline), diabetes (only baseline), educat, exercise, walk, smoke, alone.
3. As 2. but continous version of mds_cont_tri and bmi, without cross-classification.
4. As 2. examine sex heterogeneity for the variable MDS-BMI.
5. As 2. But sex-specific. Generate heatmaps.
6. Stratified analyses without cross-classification between MDS and BMI. Generate two forest plots. Treat MDS as a continuous variable, and BMI also as a continuous variable but with different HRs for BMI<26 respektive BMI>26.
7. As 2., but only age adjustment.
8. Complete case, missing at 2008/2009 means last observation carried forward.
9. As 2., but restrict the analysis to BMI>22.
10. Age-standardized descriptives.
11. Complete case (as in 8.) with additional adjustment for ”packyears”
12. As 2., but restrict the analysis to BMI≤35.
13. As 2., but do not time-update information at 2009.
14. As 2., but baseline starts in 2009.
15. As 2., but include only ”never smoker” at baseline, and censur ”ever smoker” in 2009.
