# Supplementary material for: Combined associations of body mass index and adherence to a Mediterranean-like diet with all-cause and cardiovascular mortality: A cohort study
Source: PLoS Med. 2020 Sep 17;17(9):e1003331. doi: 10.1371/journal.pmed.1003331 (PMC7497998; doi:10.1371/journal.pmed.1003331)
Supplement: S2 Protocol — BMI, body mass index (DOCX) [file pmed.1003331.s003.docx]

**S2 Protocol.** Directed acyclic graph with code (below) displaying the selection of covariates for the analysis of association of body mass index combined with adherence to a Mediterranean-like diet with mortality


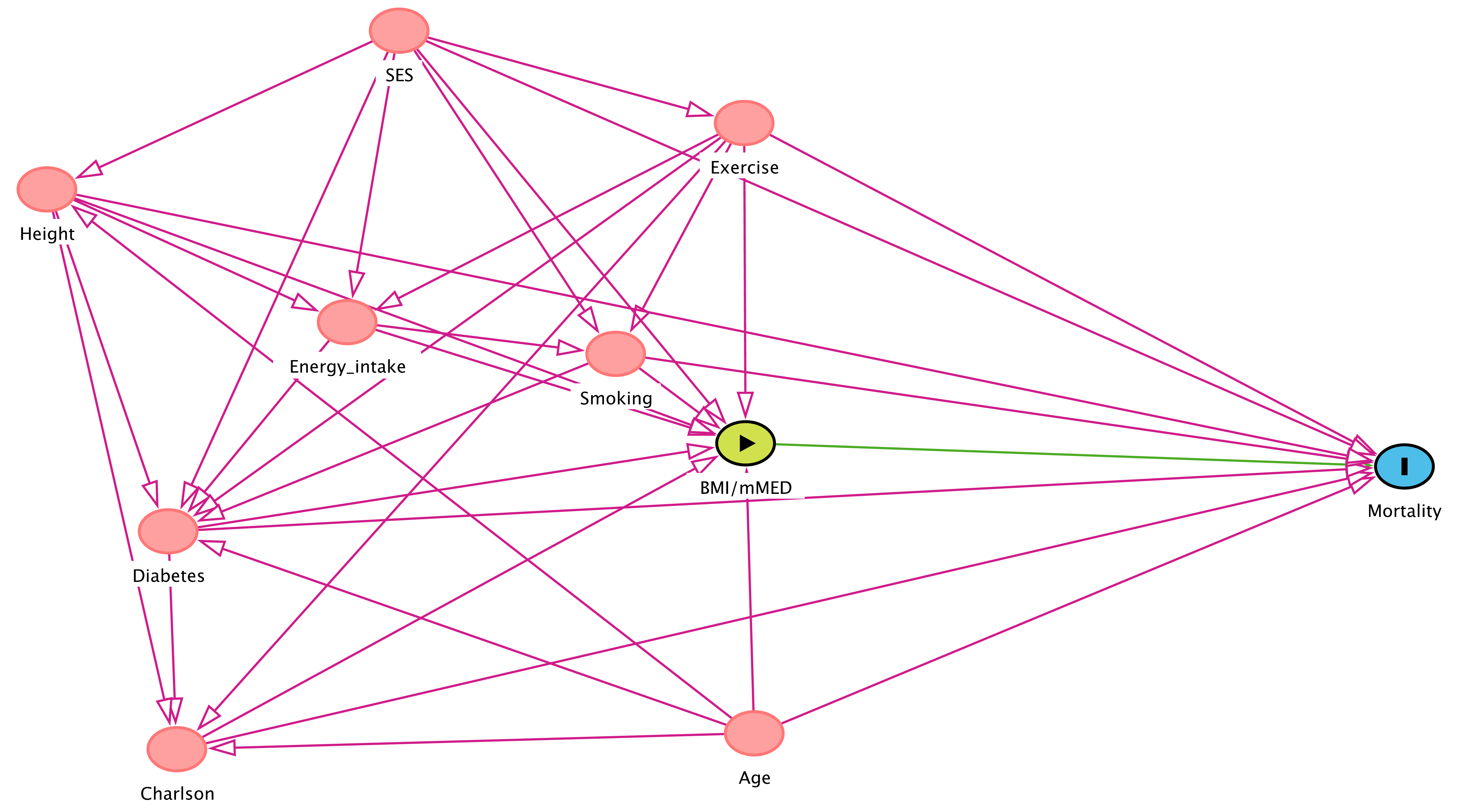


dag {

"BMI/mMED" [exposure,pos="0.461,0.445"]

Age [pos="0.467,0.817"]

Charlson [pos="0.110,0.838"]

Diabetes [pos="0.104,0.558"]

Energy_intake [pos="0.215,0.290"]

Exercise [pos="0.461,0.034"]

Height [pos="0.029,0.119"]

Mortality [outcome,pos="0.869,0.475"]

SES [pos="0.247,-0.084"]

Smoking [pos="0.381,0.331"]

"BMI/mMED" -> Mortality

Age -> "BMI/mMED"

Age -> Charlson

Age -> Diabetes

Age -> Height

Age -> Mortality

Charlson -> "BMI/mMED"

Charlson -> Mortality

Diabetes -> "BMI/mMED"

Diabetes -> Charlson

Diabetes -> Mortality

Energy_intake -> "BMI/mMED"

Energy_intake -> Diabetes

Energy_intake -> Smoking

Exercise -> "BMI/mMED"

Exercise -> Charlson

Exercise -> Diabetes

Exercise -> Energy_intake

Exercise -> Mortality

Exercise -> Smoking

Height -> "BMI/mMED"

Height -> Charlson

Height -> Diabetes

Height -> Energy_intake

Height -> Mortality

SES -> "BMI/mMED"

SES -> Diabetes

SES -> Energy_intake

SES -> Exercise

SES -> Height

SES -> Mortality

SES -> Smoking

Smoking -> "BMI/mMED"

Smoking -> Diabetes

Smoking -> Mortality

}
