# Supplementary material for: Combined associations of body mass index and adherence to a Mediterranean-like diet with all-cause and cardiovascular mortality: A cohort study
Source: PLoS Med. 2020 Sep 17;17(9):e1003331. doi: 10.1371/journal.pmed.1003331 (PMC7497998; doi:10.1371/journal.pmed.1003331)
Supplement: S2 Fig — BMI, body mass index; mMED, modified Mediterranean-like diet (DOCX) [file pmed.1003331.s005.docx]

**S2 Fig. Associations of combinations of body mass index (BMI) and adherence to a modified Mediterranean diet (mMED) with all-cause mortality after restriction of the analysis to those with normal BMI to a more narrow 22-25 kg/m2 range.**

Estimated by multivariable adjusted hazard ratios by use of Cox regression analysis with a normal BMI and high adherence to mMED as the reference. The confidence interval in each sub-panel is expressed both in numbers and as a line representing the width. HRs adjusted for sex, age (splines with two knots), educational level (≤9, 10-12, >12 years, other), living alone (yes or no), leisure time physical exercise during the past year (<1 h/w, 1 h/w, 2-3 h/w, 4-5 h/w, >5 h/w), walking/cycling (almost never, <20 min/d, 20-40 min/d, 40-60 min/d, 1-1.5 h/d,>1.5 h/d), height (splines with two knots), energy intake (splines with two knots), smoking habits (current, former, never), Charlson’s weighted comorbidity index (continuous; 1-16), and diabetes mellitus (yes/no). After this restriction, 69,745 individuals (88%) remained in the analysis with 26,600 deaths during 1,200,582 person-years of follow-up.
