# Supplementary material for: Combined associations of body mass index and adherence to a Mediterranean-like diet with all-cause and cardiovascular mortality: A cohort study
Source: PLoS Med. 2020 Sep 17;17(9):e1003331. doi: 10.1371/journal.pmed.1003331 (PMC7497998; doi:10.1371/journal.pmed.1003331)
Supplement: S5 Fig — BMI, body mass index; mMED, modified Mediterranean-like diet (DOCX) [file pmed.1003331.s008.docx]

**S5 Fig. Associations of combinations of body mass index (BMI) and adherence to a modified Mediterranean diet (mMED) with all-cause mortality excluding individuals with any of the following criteria: ever smokers, BMI below 22 kg/m2, and those with pre-existing diseases before baseline (chronic obstructive lung disease, cancer, myocardial infarction or other ischemic heart disease, heart failure, peripheral arterial disease, and stroke) and excluding the first two years of follow-up.**

Estimated by multivariable adjusted hazard ratios by use of Cox regression analysis with a normal BMI and high adherence to mMED as the reference. The confidence interval in each sub-panel is expressed both in numbers and as a line representing the width. HRs adjusted for sex, age (splines with two knots), educational level (≤9, 10-12, >12 years, other), living alone (yes or no), leisure time physical exercise during the past year (<1 h/w, 1 h/w, 2-3 h/w, 4-5 h/w, >5 h/w), walking/cycling (almost never, <20 min/d, 20-40 min/d, 40-60 min/d, 1-1.5 h/d,>1.5 h/d), height (splines with two knots), energy intake (splines with two knots), smoking habits (current, former, never), Charlson’s weighted comorbidity index (continuous; 1-16), and diabetes mellitus (yes/no). After this restriction, 27,378 individuals (35%) remained in the analysis with 9,044 deaths during 433,791 person-years of follow-up.
