# Supplementary material for: Combined associations of body mass index and adherence to a Mediterranean-like diet with all-cause and cardiovascular mortality: A cohort study
Source: PLoS Med. 2020 Sep 17;17(9):e1003331. doi: 10.1371/journal.pmed.1003331 (PMC7497998; doi:10.1371/journal.pmed.1003331)
Supplement: S1 Table — The upper part of the table presents results with use of time-updated information and 20 years of follow-up from 1997 and the lower part with use of 9 years of follow-up from 2009. The estimated associations are all multivariable-adjusted*. Absolute RDs and RRs (at 20 years and 9 years of follow-up, respectively) are calculated from the predicted survival curves based on the multivariable-adjusted Cox model. The last column of the 9 years follow-up from 2009 presents absolute RDs calculated from pseudo-observations using a GEE model with identity link. BMI, body mass index; GEE, generalized estimated equation; mMED, modified Mediterranean-like diet; RD, risk difference; RR, relative risk. (DOCX) [file pmed.1003331.s009.docx]

**S1 Table.** Associations of combinations of body mass index (BMI) and adherence to a modified Mediterranean diet (mMED) with all-cause mortality. The upper part of the table presents results with use of time-updated information and 20 years of follow-up from 1997 and the lower part with use of 9 years of follow-up from 2009. The estimated associations are all multivariable adjusted*. Absolute risk differences and relative risks (at 20 years and 9 years of follow-up, respectively) are calculated from the predicted survival curves based on the multivariable adjusted Cox model. The last column of the 9 years follow-up from 2009 presents absolute risk differences calculated from pseudo observations using a generalized estimated equation (GEE) model with identity link.

| **BMI**  **(kg/m^2^)** | **mMED**  **(score units)** | **Hazard ratio**  **(95% CI)** | **Risk difference at 20 years (95% CI)** | | **Relative risk at 20 years (95% CI)** | **Risk difference by use of pseudo-observations (95% CI)** |
| --- | --- | --- | --- | --- | --- | --- |
|  |  | **Time-updated information and 20 years of follow-up from 1997** | | | | |
|  |  |  |  | |  |  |
| **<25** | **0-<4** | 1.61 (1.48, 1.74) | -0.094 (-0.097, -0.090) | | 1.30 (1.29, 1.32) | NA |
| **<25** | **4-<6** | 1.38 (1.33, 1.44) | -0.063 (-0.061, -0.065) | | 1.21 (1.20, 1.21) | NA |
| **<25** | **6-8** | reference | reference | | reference |  |
| **25-<30** | **0-<4** | 1.39 (1.28, 1.51) | -0.064 (-0.067, -0.061) | | 1.21 (1.20, 1.22) | NA |
| **25-<30** | **4-<6** | 1.22 (1.16, 1.27) | -0.037 (-0.039, -0.036) | | 1.12 (1.12, 1.13) | NA |
| **25-<30** | **6-8** | 0.95 (0.91, 0.99) | 0.010 (0.008, 0.011) | | 0.97 (0.96, 0.98) | NA |
| **≥30** | **0-<4** | 1.50 (1.33, 1.69) | -0.079 (-0.085, -0.075) | | 1.26 (1.24, 1.28) | NA |
| **≥30** | **4-<6** | 1.33 (1.26, 1.40) | -0.055 (-0.057, -0.053) | | 1.18 (1.17, 1.19) | NA |
| **≥30** | **6-8** | 1.04 (0.97, 1.11) | -0.007 (-0.010, -0.005) | | 1.02 (1.01, 1.03) | NA |
|  |  | **No time-updated information and 9 years of follow-up from 2009** | | | | |
| **<25** | **0-<4** | 1.71 (1.50, 1.95) | -0.084 (-0.089, -0.079) | 1.42 (1.40, 1.45) | | -0.111 (-0.145, -0.077) |
| **<25** | **4-<6** | 1.41 (1.32, 1.50) | -0.051 (-0.053, -0.049) | 1.26 (1.25, 1.27) | | -0.047 (-0.059, -0.035) |
| **<25** | **6-8** | reference | reference | reference | | reference |
| **25-<30** | **0-<4** | 1.39 (1.23, 1.57) | -0.049 (-0.054, -0.046) | 1.25 (1.23, 1.27) | | -0.064 (-0.093, -0.034) |
| **25-<30** | **4-<6** | 1.21 (1.12, 1.28) | -0.027 (-0.029, -0.024) | 1.13 (1.12, 1.14) | | -0.021 (-0.032, -0.010) |
| **25-<30** | **6-8** | 0.95 (0.89, 1.01) | 0.008 (0.005, 0.009) | 0.96 (0.95, 0.97) | | 0.012 (0.004, 0.020) |
| **≥30** | **0-<4** | 1.42 (1.18, 1.72) | -0.054 (-0.060, -0.048) | 1.27 (1.24, 1.31) | | -0.064 (-0.111, -0.016) |
| **≥30** | **4-<6** | 1.30 (1.19, 1.42) | -0.039 (-0.042, -0.036) | 1.20 (1.18, 1.22) | | -0.027 (-0.042, -0.012) |
| **≥30** | **6-8** | 1.01 (0.92, 1.10) | -0.001 (-0.004, 0.002) | 1.01 (0.99, 1.02) | | 0.006 (-0.007, 0.018) |

*The multivariable models included sex, age (splines with two knots), educational level (≤9, 10-12, >12 years, other), living alone (yes or no), leisure time physical exercise during the past year (<1 h/w, 1 h/w, 2-3 h/w, 4-5 h/w, >5 h/w), walking/cycling (almost never, <20 min/d, 20-40 min/d, 40-60 min/d, 1-1.5 h/d,>1.5 h/d), height (splines with two knots), energy intake (splines with two knots), smoking habits (current, former, never), Charlson’s weighted comorbidity index (continuous; 1-16), and diabetes mellitus (yes/no).

NA; not applicable
