# Supplementary material for: Combined associations of body mass index and adherence to a Mediterranean-like diet with all-cause and cardiovascular mortality: A cohort study
Source: PLoS Med. 2020 Sep 17;17(9):e1003331. doi: 10.1371/journal.pmed.1003331 (PMC7497998; doi:10.1371/journal.pmed.1003331)
Supplement: Attachment — Submitted filename: Response PMEDICINE-D-20-00848R1_200623.docx [file pmed.1003331.s010.docx]

**We would like to thank the editorial team and the external reviewers for a thorough review of our manuscript "Combined impact of body mass index and adherence to a Mediterranean-like diet on all-cause and cardiovascular mortality: a cohort study in women and men" (PMEDICINE-D-20-00848R1). Since one reviewer wanted us to avoid causal language in the title, it is now changed to “Combined associations of body mass index and adherence to a Mediterranean-like diet with all-cause and cardiovascular mortality: a cohort study in women and men”.**

In revising the manuscript for further consideration, your revisions should address the specific points made by each reviewer and the editors. Please also check the guidelines for revised papers at <http://journals.plos.org/plosmedicine/s/revising-your-manuscript> for any that apply to your paper. In

your rebuttal letter you should indicate your response to the reviewers' and editors' comments, the changes you have made in the manuscript, and include either an excerpt of the revised text or the location (eg: page and line number) where each change can be found. Please submit a clean version of the paper as the main article file; a version with changes marked should be uploaded as a marked up manuscript.

**AU: We appreciate the reviewers’ considerable efforts to improve our manuscript and our revised manuscript is provided in a clean version and a version with marked up changes. In our response to the reviewers’ comments, the location of the changes have been indicated by page and line number.**

In addition, we request that you upload any figures associated with your paper as individual TIF or EPS files with 300dpi resolution at resubmission; please read our figure guidelines for more information on our requirements: <http://journals.plos.org/plosmedicine/s/figures>. While revising your submission, please upload your figure files to the PACE digital diagnostic tool, <https://pacev2.apexcovantage.com/>. PACE helps ensure that figures meet PLOS requirements. To use PACE, you must first register as a user. Then, login and navigate to the UPLOAD tab, where you will find detailed instructions on how to use the tool. If you encounter any issues or have any questions when using PACE, please email us at [PLOSMedicine@plos.org](mailto:PLOSMedicine@plos.org).

**AU: The figures are now produced as TIFF files in the requested format.**

Please ensure that the paper adheres to the PLOS Data Availability Policy (see <http://journals.plos.org/plosmedicine/s/data-availability>), which requires that all data underlying the study's findings be provided in a repository or as Supporting Information. For data residing with a third party, authors are required to provide instructions with contact information for obtaining the data. PLOS journals do not allow statements supported by "data not shown" or "unpublished results." For such statements, authors must provide supporting data or cite public sources that include it.

**AU: Results from sensitivity analyses previously not displayed are now shown in the supplementary information.**

Requests from the editors:

*In the last sentence of the Abstract Methods and Findings section, please describe the main limitation(s) of the study's methodology.

**AU: This is now described as “A main limitation of the present study is the observational design. No causal inferences should be made based on this study alone”.**

*Due to the observational design of the study, we'd suggest care in using causal language - at the moment the abstract uses language such as "reduce(s) risk..", which may be better phrased as "associated with a reduction in risk". There may be other places to modify this too.

**AU: In the revised version of the manuscript, we have removed the causal language at all places.**

*Did your study have a prospective protocol or analysis plan? Please state this (either way) early in the Methods section.

**AU: Yes, we used a prospective statistical analysis plan (in Swedish) and this file is now provided in the Supporting information data file.**

a) If a prospective analysis plan (from your funding proposal, IRB or other ethics committee submission, study protocol, or other planning document written before analyzing the data) was used in designing the study, please include the relevant prospectively written document with your revised manuscript as a Supporting Information file to be published alongside your study, and cite it in the Methods section. A legend for this file should be included at the end of your manuscript.

**AU: A pre-specified analysis plan in Swedish can be found at:** <https://www.protocols.io/edit/body-mass-index-healthy-diet-and-mortality-bgftjtnn>

b) If no such document exists, please make sure that the Methods section transparently describes when analyses were planned, and when/why any data-driven changes to analyses took place.

**AU: Not applicable**

c) In either case, changes in the analysis-- including those made in response to peer review comments-- should be identified as such in the Methods section of the paper, with rationale.

**AU: All changes in the analysis as recommended in response to peer review comments are highlighted as such in the Methods.**

*We'd suggest ensuring that the study is reported according to the STROBE guideline, and include the completed STROBE checklist as Supporting Information. Please add the following statement, or similar, to the Methods: "This study is reported as per the Strengthening the Reporting of Observational Studies in Epidemiology (STROBE) guideline (SChecklist)." The STROBE guideline can be found here: <http://www.equator-network.org/reporting-guidelines/strobe/>. When completing the checklist, please use section and paragraph numbers, rather than page numbers.

**AU: A STROBE checklist has been included in the Supporting information**

-----------------------------------------------------------

Comments from the reviewers:

Reviewer #1: Statistical review

This paper reports the analysis of a cohort study investigating association between diet (as quantified with a Mediterranean diet score) and BMI on mortality. The follow-up is long and the sample size is high; I also thought generally the methods and reporting was good. I have some minor comments:

1. Abstract - "Obese individuals with high mMED did not experience

higher rates of all-cause mortality" - add something along the lines of 'there was no significant evidence of..' or 'did not experience significantly higher…'

**AU: Changed as suggested**

2. Given the questionnaire completion was moderate, especially in men, can anything be added on how representative those who completed the questionnaire are of the general population?

**AU: Information regarding representativeness is now provided on p 6, line 12-14.**

3. Page 9 "Covariates, chosen using directed acyclic graphs," - can more information be given on how they were selected? Did this use any data?

**AU: The DAG and code can now be found in the Supporting information and the information about the DAG in the manuscript is provided on p 11, line 2. We used no data analysis to draw the DAG.**

4. Page 9 -how much missing data was there was on the covariates (it would be sufficient to say what proportion were complete cases)?

**AU: The degree of missingness is now described on p 11: The proportion of missing data in the cohorts was 4% for BMI, 3% for height, 9% for walking/bicycling, 11% for exercise, and 6% for marital status. For all other covariates the percentage of missing was less than 2%. Missingness of foods at baseline was for fruit and vegetables 0.1%, legumes and nuts 3.4%, grains 0.9%, fermented dairy products 2.9%, fish 1.1%, meat 0.7%, olive or rapeseed oil 0%, and alcohol intake 0%.**

5. Page 9 - "We performed additional analyses in subgroups of potential confounders in which BMI below or above the median of 26 kg/m2 and mMDS were included as continuous variables.": I didn't follow this; what were the confounders, were they pre-specified and is this a stratified analysis or something different? It would be useful if an analysis plan was included if this was pre-specified.

**AU: Yes, this is a stratified analysis displaying HRs per unit change in BMI and MDS by categories of the prespecified confounders, as described in our analysis plan. This is the pre-planned analysis: No 6 in the analysis plan.**

6. Page 11 - the results of the sensitivity analyses should be provided in supplementary material (as per PLOS medicine guidance prohibiting 'data not shown').

**AU: Sorry, this was overlooked by us. The results are now provided in the Supporting information.**

James Wason

-----------------------------------------------------------

Reviewer #2: This well-written manuscript presents the results of individual analyses of BMI and a Mediterranean diet score, as well as a joint analysis of these, in relation to all-cause and CVD mortality in a Swedish cohort of older adults. The work has many strengths; here I will detail some areas in which I believe that it could be improved. In particular, I have grappled with what the purpose of the study is, what the results may be telling us, and what they can be used for.

Major comments:

1. The authors present a 3x3 table, detailing a joint analysis of two exposures on the multiplicative scale. This is in essence an interaction analysis, and I commend it. It appears that there is no interaction between BMI and diet quality, and that diet quality alone associates with greater all-cause mortality. The authors conclude that it is more important to focus on improving diet quality than preventing obesity to lower all-cause mortality in this age group. However, the individual analyses of BMI and diet show that each of these exposures are associated with all-cause mortality. In the absence of a multiplicative interaction, there must be an interaction on the additive scale. What are the implications of this for public health messages? Ought public health interventions to be targeting diet alone, which the conclusions of the abstract indicate should be the case? Where can most lives be saved - among those with poor diets or those who are of an unhealthy BMI (see also

comment below)? If this is the purpose of the study, I would recommend redoing the analyses using methods that estimate risk differences. The pseudoobservation method can be used for time-to-event data; others also exist. (see <https://doi.org/10.1186/1471-2288-14-97> ; <https://doi.org/10.1515/em-2017-0015>) If the intention is to assess multiplicative interactions, please be mindful of how these are greatly dependent on the baseline hazard of death, and consider this in the interpretation of the results. Also, the effects of time varying exposures on time-varying confounders, and any feedback loops between these, cannot be estimated without bias using Cox proportional hazards. In this case, g computation is preferable, as this bias can be taken into account.

**AU: We thank the reviewer for the valuable and thorough review. Interaction, or absence of interaction on the multiplicative scale may indeed indicate that there is interaction on the additive scale and we agree that risk differences would add more information with regards to the public health message. That said, no public health recommendations should of course be based on one single study.**

**We have gone through the references of the pseudo observation method kindly provided by the reviewer and also several additional ones. For example, Östergaard et al from 2016 states that time varying covariates can not be included, which is a short coming of the method. Although we use time-updated information on several of the covariates and for the exposure, we tried the pseudo-observation method but the estimated survival curves could be increasing after the update, which makes no sense. Instead we used the method outlined in Austin et al from 2010 to estimate adjusted risk differences, S_exp_j_(t=20) – S_ref_(t=20), where S_ref_ is the predicted survival probability for the reference category and S_exp_j_ the predicted survival probability for the exposure category j, for all-cause mortality estimated based on the multivariable Cox model with use of time-updated information. The adjusted relative risks, (1- S_exp_j_(t=20))/ (1- S_ref_(t=20)), were calculated using the same method. The results are presented in S1 Table.**

**Notably, regarding reference to time varying exposures and time-varying confounders with possible feedback loops as mentioned by the reviewer, our results were quite similar if we used 2009 and the second examination as the baseline for our analysis. Multivariable adjusted HRs for all-cause mortality by combinations of BMI and eMDS are displayed in the figures below. The adjusted relative risks and the adjusted risk differences were calculated at 9 years of follow-up with the same approach as mentioned above (with results provided in the lower part of S1 Table). In addition, as there were no time updated covariates in this data set, we also estimated the risk differences as suggested by the reviewer and as outlined in Ambrogi et al, using the pseudo observations as the outcome variable in a GEE model with the identity link function. The results follow the same pattern as the RD from the Cox model but with wider confidence intervals (lower part of S1 Table).**

**Heat map of multivariable adjusted HRs for all-cause mortality by combinations of BMI and mMDS using the second examination in 2009 as baseline:**

**Heat map of multivariable adjusted HRs for cardiovascular mortality by combinations of BMI and mMDS using the second examination in 2009 as baseline:**

2. The current conclusion of the paper, and in particular the abstract, is based on the results for all-cause mortality. The results for CVD mortality are not entirely in agreement, and given that CVD-deaths are over 1/3 of the total number of deaths, the conclusions should take these results into account. Some further discussion of the possible other important causes of death for this age group would also be useful. Are the results, which indicate that BMI is not associated with these other causes of death for a given diet quality, reasonable?

**AU: We agree that the CVD mortality findings differ in some ways from those for all-cause mortality, our primary outcome. The wording of the final sentences of the Abstract is now changed to: “These findings suggest, that for older women and men, diet quality modifies the association between BMI and all-cause mortality. A healthy diet may, however, not completely counter higher CVD mortality related with obesity.”**

**In addition to previously suggested mechanisms, we have now (p19, last sentence of Potential mechanisms in the Discussion) described with references “Concomitant low body mass index and malnutrition, a nutritional state in which nutrient intake does not match nutrient needs, could in elderly individuals also lead to decreased immune function followed by higher risk of infections [67, 68] and higher risk of surgical complications [69], including a fourfold greater risk of mortality and more frequent re‐admissions [70].”**

3. Further discussion of the importance of the different body compartments that BMI collectively measures in this age group would be very helpful in interpreting the results, particularly in this context of a very long follow up. The brief discussion of sarcopenia is interesting, but how does this relate to the normal BMI category? Would these participants be considered sarcopenic and frail? What are the chances that some of the obese participants actually have a high muscle mass for their age, and that this is protective? What might be considered a healthy or an unhealthy BMI in this age group, and why? While the authors explicitly state that the study cannot tell us about changes in BMI or diet quality over time, the data are available to cast some light on this…

**AU: We have in a subcohort (n=5022) of the women data on body composition measured by DXA (Lunar Prodigy), measured in 2003-2009. Low lean mass (appendicular lean mass index <5.45 kg/m^2^ according to Baumgartner’s definition from 1998) at mean age of 68 years was present in only 6% of the women. BMI was highly correlated with total fat mass (r=0.92) and total fat mass index (r=0.95) while the correlation between BMI and lean mass was 0.49 and with lean mass index was 0.68. Long-term change of BMI in our cohort of women was more strongly correlated with fat mass (r=0.62) than with lean mass (r=0.24). A detailed analysis of body composition is not within the scope of the present analysis but we appreciate the suggestions from the reviewer, which could be a theme for future analysis. The prevalence of sarcopenia at baseline in our larger cohorts is not known.**

**The definition of a healthy BMI at different ages is not recommended to be decided by our single study. Our result might rather be considered as one scientific piece in a complicated research puzzle dealing with BMI, and our message is that BMI may not be considered in isolation but rather in conjunction with dietary habits.**

Minor comments:

1. Please be mindful of the terms used to describe the results. For example in a couple of places, the manuscript mentions presenting rates, but the results are hazard ratios (ie a relative measure of association).

**AU: The usage of the word rate in the Results has been replaced by hazard ratio (or HRs).**

2. Please consider including the DAG used to select confounders in the supplements. For instance, I am curious how diabetes can be a (time-varying?) confounder, when this disease is partially caused by elevated BMI and poor dietary habits. I would think that diabetes was an intermediate variable, and that adjusting for this would cause bias of the associations. I am also curious as to why height is considered a confounder.

**AU: We thank the reviewer for providing us with this point of concern. The presentation of diabetes and comorbidity index as time-varying covariates was an incorrect description on our part. We apologize for this mistake and the ensuing confusion. Only baseline values of these variables were considered in the multivariable analysis and this has been clarified. We definitely agree that new cases of diabetes and changes in comorbidity during follow-up should mainly be regarded as intermediates.**

3. The purpose of the analyses presented in figure 2 is not clearly described, and the results are not interpreted in depth. Please either justify these analyses more clearly, or omit them.

**AU: The use of Figure 2 has several explanations. Both BMI and dietary results are commonly discussed in relation to different types of biases and confounding and we found it valuable to present these exposures within strata of the covariates. We thought it would be somewhat re-assuring for the reader to see that the associations were in the same direction within all subgroups of the covariates. For example smoking habits and low socioeconomic status have previously been discussed as underlying causes for these associations but as the reader can see, a similar pattern for the associations between both BMI with mortality and MDS with mortality within categories of smoking status, educational level and marital status. Moreover, displaying results within strata of confounders is a recommended approach according to STROBE guidelines. Ideally, the presentation of our main exposure associations within strata of confounders would have been preferable but with nine combined categories of BMI and MDS this would have rendered low precision of the estimates and also leading to a risk of a too complicated message.**

4. The test used to derive the p for homogeneity is not described in the methods section.

**AU: A reference to the method by Fleiss is now provided.**

-----------------------------------------------------------

Reviewer #3: Michaëlsson et al evaluated the combined associations of BMI and adherence to a Mediterranean-like diet on all-cause and CVD mortality among men and women in the Swedish Mammography Cohort and the Cohort of Swedish Men. When analyses were cross-classified by categories of BMI and mMED score, the authors identified the lowest rates of all-cause mortality among overweight individuals with high mMED compared with those with normal weight and high mMED. At the same time, obese individuals with high mMED did not experience higher rates of all-cause mortality while individuals with a low mMED had a high mortality despite a normal BMI. This is a well-done, interesting, and important analysis highlighting the modifying effect of diet. However, there are major methodological considerations outlined below that need to be adequately addressed:

1. Throughout the manuscript, please avoid use of causal terminology (eg: reduced replace with "lower") given the observational nature of the study. Along these lines, it is recommended that the authors replace the term "impact" in the title to a non-causal term such as "associations".

**AU: We would like to thank the reviewer for a careful evaluation. We have now changed the wording, avoiding causal terminology.**

2. This study does not aim to compare the relative effectiveness of lowering BMI versus a higher diet quality. Therefore, the "conclusion" in the abstract and the "meaning" in the key points section should reflect what the study actually did - that diet quality "modifies" the higher risk seen with higher BMI.

**AU: The concluding sentences of the abstract have been reworded to: These findings suggest, that for older women and men, diet quality modifies the association between BMI and all-cause mortality. A healthy diet may, however, not completely counter higher CVD mortality related with obesity.**

3. Replace the term "subjects" with "participants".

**AU: Changed as suggested by the reviewer.**

4. Respiratory diseases (such as COPD and pulmonary disease) are the major causes of low BMI. Did the authors consider excluding these individuals?

**AU: As recommended, we have now performed an additional sensitivity analysis that excluded those with prevalent chronic pulmonary disease, please see below under the last item from the reviewer.**

5. Several studies have shown that confounding due to smoking is strikingly strong in analyses of BMI and mortality and that complete elimination of confounding is only possible by restricting to never smokers. The current analysis remains severely confounded by smoking status which may partly explain the higher HR's in the normal BMI range compared to the obese BMI range. The authors should attempt to carefully and completely control for confounding due to smoking status.

**AU: In the main analyses, by adjusting for smoking status, we could theoretically overestimate the biological effect of low BMI due to residual confounding by smoking but another view can be that this model rather evaluates the association of MDS and BMI with mortality on a whole population level (in a mix of never, former and current smokers) with a consequence of better external validity.**

**Nevertheless, analysis restricted to never smokers has been conducted and the results are now displayed in SFigure4. The pattern of the HRs was similar compared with an analysis without this restriction, as shown in SFigure4, although the restriction excluded more than 40% of the participants. Moreover, the associations between BMI and mortality and the association between mMED and mortality were quite similar within categories of smoking status (Figure 2).**

6. Given that pack-years of smoking was available, it is not clear why this was not adjusted for in the primary analysis. Residual confounding due to smoking is inevitable, it is critical to adjust for this as completely as possible.

**AU: In S3 Fig, we display the results after adding pack-years of smoking to the multivariable model. Again, the results remained similar. A conceivable problem with the use of pack-years is both the possible inaccurate retrospective collection of smoking habits, several decades before the baseline, and that pack-years of smoking itself is a mixture of dose, recency of use and duration of use, confounding current smoking status, duration and amount of smoking.**

7. For CVD mortality analysis, did the authors exclude those with CVD at baseline?

**AU: No, we did not since our primary aim was to evaluate associations on a population level and CVDs can be recurring events. Instead, we adjusted for baseline differences in comorbidity.**

8. The "normal" range of BMI consists of a very heterogenous group of participants including chronic smokers (see higher % of current smokers in the normal BMI category in Table 1), those with preexisting conditions, and healthy individuals. Although excluding individuals with a BMI <20 kg/m2 excludes to some extent the reverse causation due to preexisting diseases, any analyses examining BMI and mortality should consider all the following exclusions/restrictions simultaneously - 1) restrict analyses to never smokers, 2) exclude those with preexisting disease (including respiratory disease and CVD and not just cancer), 3) and exclude deaths during the first few years of follow-up. While the authors attempted to do some of these in sensitivity analyses (data not shown), it is not clear if these were done concurrently.

9. It is recommended that the authors present analyses restricted to never smokers, normal BMI restricted to 22-25 kg/m2, excluding the first two years of follow-up and those with pre-existing diseases (respiratory diseases, cancer, CVD) as the primary analyses.

**AU: In response to comments 8 and 9, we have performed a massive restriction as suggested: (restriction to never smokers, excluding BMI below 22 kg/m2, and those with pre-existing diseases before baseline [chronic obstructive lung disease, cancer, myocardial infarction or other ischemic heart disease, heart failure, peripheral arterial disease, and stroke], leaving only 35% of our original participants remaining for this sensitivity analysis and in addition excluding the first two years of follow-up. Still, the pattern of the HRs for all-cause mortality is comparable (S5 Fig) as those from the main original model without the restrictions.**
